# Probiotic induced synthesis of microbiota polyamine as a nutraceutical for metabolic syndrome and obesity-related type 2 diabetes

**DOI:** 10.3389/fendo.2022.1094258

**Published:** 2023-01-13

**Authors:** Tina I. Bui, Emily A. Britt, Gowrishankar Muthukrishnan, Steven R. Gill

**Affiliations:** ^1^ Department of Microbiology and Immunology, University of Rochester Medical Center, Rochester, NY, United States; ^2^ Center for Musculoskeletal Research, University of Rochester Medical Center, Rochester, NY, United States; ^3^ Department of Orthopedics, University of Rochester Medical Center, Rochester, NY, United States

**Keywords:** polyamine, obesity, type 2 diabetes, metabolic syndrome, nutraceutical

## Abstract

The gut microbiota regulates multiple facets of host metabolism and immunity through the production of signaling metabolites, such as polyamines which are small organic compounds that are essential to host cell growth and lymphocyte activation. Polyamines are most abundant in the intestinal lumen, where their synthesis by the gut microbiota is influenced by microbiome composition and host diet. Disruption of the host gut microbiome in metabolic syndrome and obesity-related type 2 diabetes (obesity/T2D) results in potential dysregulation of polyamine synthesis. A growing body of evidence suggests that restoration of the dysbiotic gut microbiota and polyamine synthesis is effective in ameliorating metabolic syndrome and strengthening the impaired immune responses of obesity/T2D. In this review, we discuss existing studies on gut microbiome determinants of polyamine synthesis, polyamine production in obesity/T2D, and evidence that demonstrates the potential of polyamines as a nutraceutical in obesity/T2D hosts.

## Introduction

More than 90% of diabetes is attributed to obesity and it is therefore referred to as obesity-related type 2 diabetes (obesity/T2D) (1). The majority of individuals with obesity/T2D are also diagnosed with metabolic syndrome, based on diagnostic criteria that includes increased waist circumference (i.e., central obesity), elevated triglycerides, and abnormally high fasting glucose indicative of insulin resistance (2, 3). Based on predictions from 2007, the prevalence of diabetes is expected to reach 366 million worldwide by 2030 (4). However, recent epidemiological findings suggest 537 million individuals were already affected by obesity/T2D globally in 2021 (5), surpassing the original 2007 estimate (4). Parallel increases in obesity and type 2 diabetes are major public health concerns as both are associated with lower health-related quality of life (6, 7) such as adverse post-infection clinical outcomes (8–12). For example, both obesity and diabetes increase the risk for post-operative periprosthetic joint infections (PJIs) (9) and surgical site infections (SSIs) (10). During the COVID-19 pandemic, individuals with obesity/T2D were more likely to be hospitalized (11, 12) with some studies suggesting higher in-hospital mortality rates (13) likely due to increased incidence of cytokine storms preceding septic shock (12). Thus, obesity/T2D is linked to deficits in overall immunity that are associated with severe infections across body sites at higher mortality and morbidity rates compared to non-obese/T2D counterparts. This in part occurs due to chronic low-grade inflammation that is associated with a disturbance in gut microbiome composition and metabolism as compared to a healthy or homeostatic baseline known as gut dysbiosis (14–22).

It is well accepted that long-term exposure to prototypical Western diets characterized by highly processed foods is at the core of obesity/T2D-related metabolic syndrome (23–25) and chronic low-grade inflammation (26). The gut microbiome as the mediator linking diet and obesity has been established in multiple human and animal studies, with diet identified as the primary contributor to changes in gut microbiome diversity and functional capacity associated with obesity and impaired immunity (27–29). Metabolomic studies have identified several groups of gut microbiota metabolites associated with obesity/T2D, including short-chain fatty acids (30–33), triethylamine-N-oxide (34–36), and bile acids (30, 37–39), which regulate host metabolism and immunity. In this review, we focus on polyamines, a group of metabolites increasingly associated with obesity/T2D.

Polyamine metabolites are essential to mammalian health in multiple organs (40) where they regulate cellular metabolism, proliferation, and differentiation (40, 41). Despite extensive work on polyamines in cancer, autoimmune diseases, and aging, the role of polyamines in immunity remains obscure. More importantly, the role of polyamines in obesity/T2D immunity is not well-studied. Herein, we review the contribution of polyamine biosynthesis and function to obesity/T2D, host immunity, and evaluate new work that utilizes polyamines to mitigate metabolic syndrome and complications of obesity/T2D. Understanding the role of polyamines on immunity and metabolic diseases will lead to identification of novel, alternative therapeutics for immunocompromised obese/T2D patients.

## Polyamine and obesity-related type 2 diabetes

### The gut microbiota is a major contributor to the host polyamine pool

**Figure 1:**
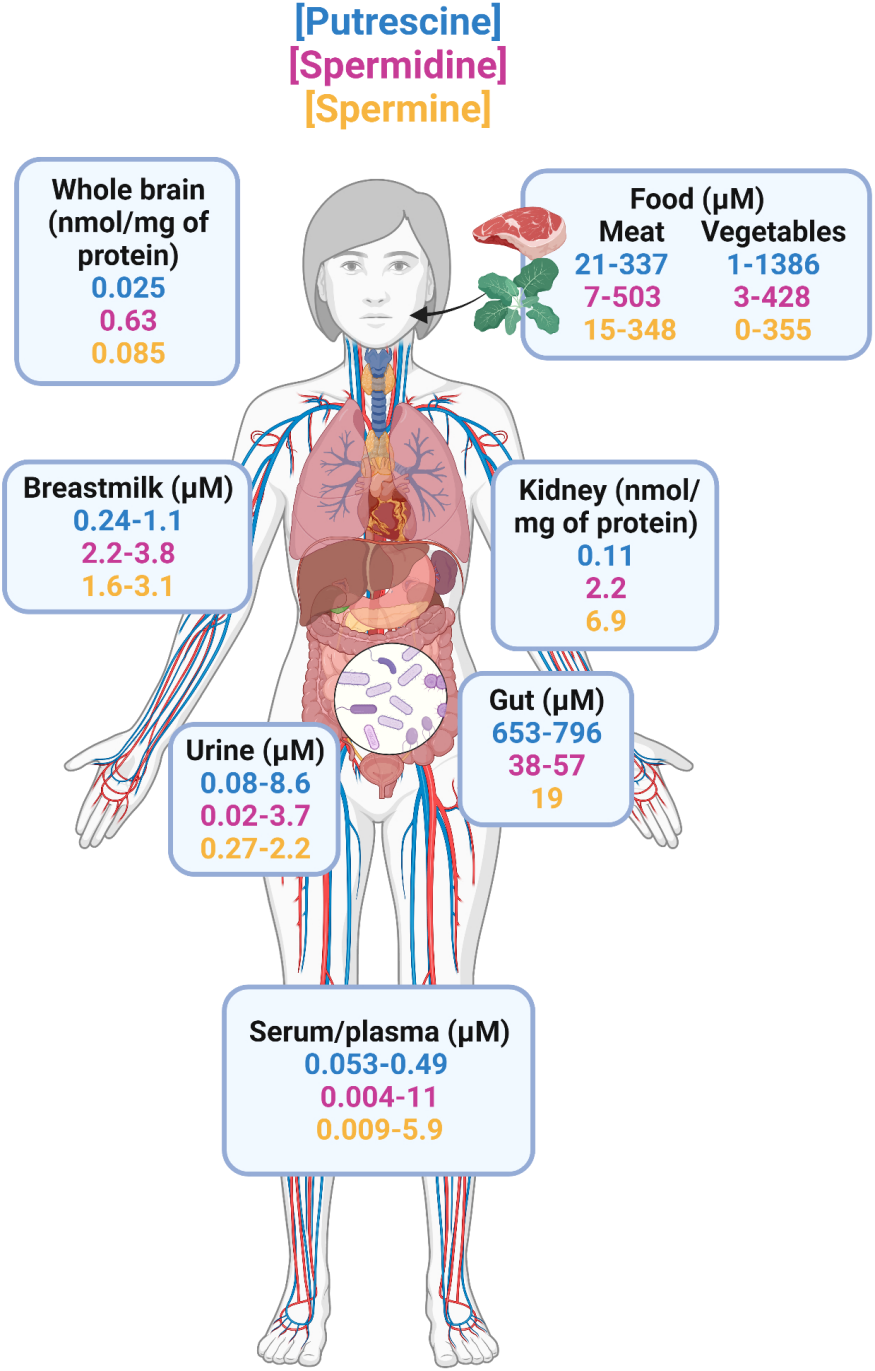
Distribution of natural polyamines in healthy adults. The three most abundant polyamines in humans are putrescine, spermidine, and spermine. Total polyamine is highest in the intestine because diet and gut microbiome are the primary sources of polyamines. From the gastrointestinal tract, polyamines are readily absorbed and enter circulation where they are found in a variety of organs including the brain, kidneys, and in metabolic fluids such as urine, and breastmilk. Studies that reported concentrations in ng/mL or μg/mL were transformed to μM using the molecular weights of putrescine (88.15 g/mol), spermidine (145.25 g/mol), and spermine (202.34 g/mol) for better comparisons.

Polyamines are produced primarily from amino acid precursors arginine, ornithine, and methionine (42). Diamine putrescine, triamine spermine, and tetraamine spermidine are the most abundant natural polyamines found in mammals (43). The total polyamine reservoir in mammals is regulated endogenously by host cells (43) and exogenously by gut bacteria (44–47) and diet (48, 49). Putrescine, spermidine, and spermine exist in the micromolar to millimolar range in various foods (48, 49) and across body sites of healthy adults as summarized in **Figure 1**. The use of radiolabeled polyamines demonstrated that polyamines are readily absorbed and enter the circulation (48), where they are distributed across different organs and metabolic fluids, including the brain (50), kidneys (51), breastmilk (52–54), urine (55–58), and serum/plasma (55, 56, 59–62). Notably, the largest accumulation of polyamines occurs in the intestinal lumen as determined with fecal samples from healthy donors where putrescine is the most abundant followed by spermidine and spermine (63, 64). Fecal levels of polyamines have been shown to correlate with gut microbiota composition in humans (63–65) and rodent models (66, 67). Oral supplementation in rodents with arginine led to increased polyamine concentrations in feces in a dose-dependent manner, which was eliminated when these animals were treated with antibiotics prior to arginine administration (66). This implicates the role of the gut microbiota in amino acid metabolism that results in synthesis of polyamines in mammals. Additionally, both Gram-positive and Gram-negative bacteria isolated from human fecal samples, including *Bifidobacterium*, *Clostridium*, *Enterococcus*, and *Lactobacillus*, are able to produce polyamines *in vitro* (68). Of these genera, the abundance of *Bifidobacterium animalis* is decreased and *Lactobacillus reuteri* increased in obese individuals (69, 70). Together, these studies demonstrate that the gut microbiota is a major contributor to the total polyamine pool *in vivo*. Moreover, with the direct link between obesity/T2D and gut dysbiosis (71, 72), there is a compelling and urgent need to study host-microbiota-polyamine interactions and the contribution of polyamines in obesity/T2D.

### Polyamine synthesis in obesity/type 2 diabetes

Thus far, there are only a handful of studies that have reported polyamine levels in obese individuals, all of which examined circulating polyamines in serum or plasma (73–75). In a study of 114 overweight/obese adults (BMI=27-40 kg/m^2^) with and without type 2 diabetes, it was concluded that individuals with obesity/T2D had elevated levels of putrescine as compared to obese, non-diabetic participants (73). This study did not have an additional control group of healthy adults but levels of putrescine (0.076μM), spermidine (0.15μM), and spermine (0.027μM) were in range when compared to previously reported levels in plasma/serum of healthy adults (putrescine = 0.053-0.49μM, spermidine = 0.004-11μM, spermine = 0.009-5.9μM) (55, 56, 59–62). In a second cohort of morbidly obese adults, the same group of authors noted increases in serum putrescine that was associated with failure to ameliorate metabolic syndrome six months after bariatric surgery (74). These results differ from a third study of 102 obese adults that demonstrated there were no differences circulating levels of putrescine, spermine, and spermidine in male vs female obese adults (76). Unfortunately, this study also did not have healthy adult controls but when compared to studies in healthy adults, only putrescine was elevated whereas spermine and spermidine were in the range of previously reported levels (55, 56, 59–62). More recently, spermidine was found to be positively associated with obesity in rural Chinese residents (75). However, these individuals were more likely to decrease their BMI over two years in a follow-up study (75). This suggests that spermidine exert protective effects against weight gain but of unknown mechanism. Large ranges can be seen across these studies illustrated in **Figure 1**. Variation in these studies likely originates from: i) different methods used to extract and analyze polyamines; e.g., HPLC was used in all obese/T2D studies whereas LCMS is considered to be more accurate and sensitive, ii) only including male participants (59), iii) not considering potential correlation between age and polyamines that has been previously identified (40, 63, 77). Therefore, although the majority of existing studies suggest polyamines are elevated in obesity/T2D, it is difficult to make substantial conclusions without control non-obese, non-diabetic healthy adults because exogenous polyamines have anti-obesity effects in preclinical studies (78–81). Additionally, patients with type 2 diabetes expressed reduced levels of polyamine synthetic enzymes such as ornithine decarboxylase that is necessary for putrescine production (82). Because these studies suggest an elevation in only putrescine when compared to healthy adults of other studies, it is important to identify changes in individual polyamine metabolites in future works. Note that these trends were also concluded from studies primarily in European or rural Chinese residents (59, 73–76). Additional studies are required in countries where obesity/T2D affect over 20% of the population including the United States, Mexico, Russia, and Brazil (83, 84).

Results from animal models of obesity-related diseases are also difficult to interpret due to the use of heterogenous models of obesity/T2D and sampling of tissues. Adipocytes from obese Zucker rats demonstrated a 4-fold increase in concentration of spermine and spermidine that was positively associated with adipose triacylglycerol formation (85). However, two-month-old leptin-deficient obese/T2D mice exhibited 31% less spermidine but 24% higher spermine in whole pancreatic islets (86). Obesity/T2D decreased expression of polyamine synthetic genes *Odc* (ornithine decarboxylase)*, Srm* (spermidine synthase), and *Sms* (spermine synthase) that concomitantly resulted in a ~30% reduction in spermidine in the colon (87). These results differed from another study in diet-induced obese/T2D murine model that demonstrated no deficiency in polyamines due to obesity/T2D locally in the gut or systemically in plasma (88). Despite the variations in both humans and animal models of obesity/T2D, polyamines are essential to white adipose tissue homeostasis by stimulating adipocyte lipolysis (81) and the deletion of a spermidine to spermine conversion enzyme, spermidine/spermine N1-acetyltransferase, leads to late-onset obesity and insulin resistance (89–91).

### Polyamines regulate host immunity

Polyamines regulate a variety of cellular functions in both innate and adaptive immune cells that are often described to be immunosuppressive (41). At neutral pH, polyamines exist as positively charged molecules that interact with negatively charged macromolecules such as DNA, RNA, and proteins (43). As a result, polyamines are necessary for molecular regulation of growth, autophagy, differentiation, and activation of lymphocytes. Polyamine depletion with enzyme inhibitors induced abnormal differentiation of cytolytic T lymphocytes (92) and caused defects in B-cell production of immunoglobulins (93). Both of these phenotypes were rescued upon exogenous addition of polyamines. T helper cells are the most well-studied when investigating the role of polyamines. Polyamines regulate CD4+ T cell differentiation as demonstrated by spermidine induction of *Foxp3* expression to polarize naïve T cells to become regulatory T cells in an autophagy-dependent manner (94). In addition to Tregs, polyamines have been proposed to regulate transcription factors such as *Tbx21* (T-Bet), *Gata3*, and *Rorc* that mediate CD4+ T cell differentiation into other subsets Th1, Th2, and Th17 epigenetically (95). This suggests that polyamines have significant impacts on adaptive immunity.

The role of polyamines in innate immune cells are less clearly defined. Polyamine synthesis is required for natural killer cell metabolism and effector function including the production of granzyme B and IFN-γ (96). In neutrophils, polyamines regulate effector functions by promoting superoxide and myeloperoxidase production. In healthy human neutrophils, depletion of polyamines treatment led to decreased production of superoxides ( ${\text{O}}_{2}^{-}$ , H_2_O_2_) and release of myeloperoxidase (97). This is congruent with a second study demonstrating physiological concentrations of spermidine induced superoxide generation in human neutrophils stimulated with chemotactic peptide fMet-Leu-Phe (98). While the effects of polyamines are pro-inflammatory in natural killer cells and neutrophils, polyamines are immunosuppressive in monocytes/macrophages. Spermine inhibited synthesis of pro-inflammatory cytokines (TNF, IL-1, IL-6, MIP-1α, MIP-1β) in LPS-stimulated human peripheral blood mononuclear cells (99) and inhibited the production of nitric oxide in J774 murine macrophages stimulated with LPS or IFN-γ (100). Spermidine promoted hypusination of the eukaryotic translation factor eIF5A that switches macrophage metabolism to oxidative phosphorylation, a phenotype associated with M2-like anti-inflammatory macrophages (95, 101). However, depletion of polyamines in the same cell line induced nitric oxide synthesis when macrophages were stimulated with LPS (102). Thus, the exact role of polyamines in immune cells, particularly innate immune cells, remains unclear. It is particularly interesting to investigate the role of polyamines in innate immune cell dysfunction in obesity/T2D.

## Polyamines as a nutraceutical

### Polyamines as a nutraceutical for metabolic syndrome in obesity/T2D

Several studies have demonstrated beneficial effects of increased polyamine concentrations in correcting the metabolic complications of obesity/T2D (78–81, 87, 103). The total host polyamine pool can be increased either with diet (**Figure 1**) or by supplementation with probiotics or prebiotics which increase the abundance of gut bacteria that synthesize polyamines (44–47, 68). *Bifidobacterium* spp. are often regarded as beneficial bacteria due to their production of short-chain fatty acids, which has a myriad of physiological effects on the host (104). More recently, *Bifidobacterium* spp. have also been linked to polyamine synthesis in animal models of obesity/T2D and importantly, improved clinical outcomes (67, 87, 88). In a diet-induced model of obesity/T2D, mice fed *Bifidobacterium animalis* subsp. *lactis* for 12 weeks rescued polyamine production with a concomitant correction of lipid and glucose metabolism, reduction in metabolic endotoxemia, and strengthening of gut barrier function (87). Similarly, administration of *B. lactis* with arginine, an amino acid precursor to polyamine synthesis, resulted in increased levels of circulating and colonic levels of polyamines that correlated with reduced inflammation in senescent mice (67). Furthermore, in another diet-induced obese/T2D mouse model, supplementation with oligofructose, a bifidogenic indigestible carbohydrate, led to increases in abundance of *B. pseudolongum* that was associated with elevation of bacteria-specific polyamine precursor acetyl-ornithine and down-stream levels of spermine and spermidine (88).

**Figure 2:**
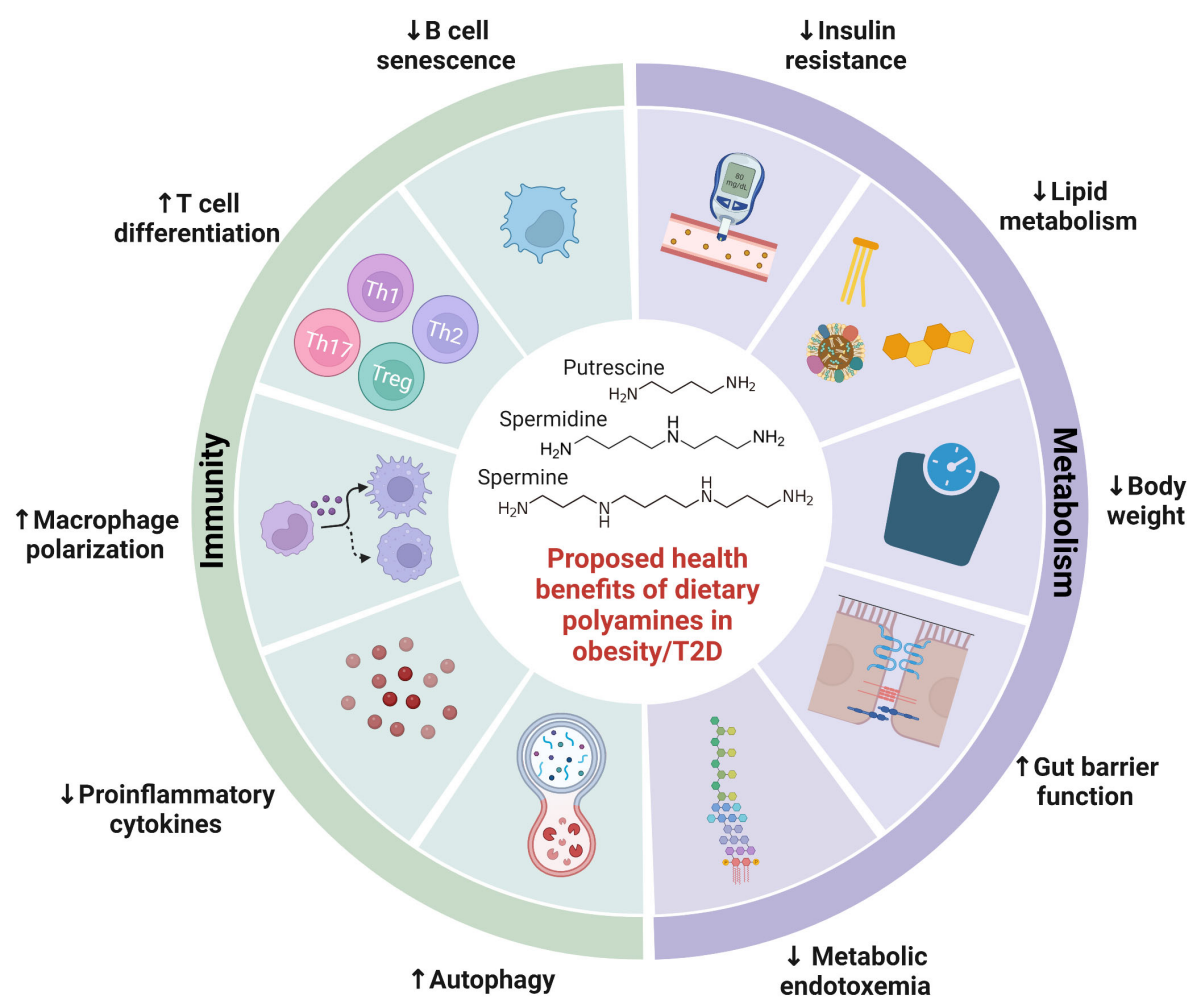
Proposed health benefits of dietary polyamines in obesity-related diseases. Polyamines are essential to human health, with their abundance altered by obesity-related diseases. This figure highlights a summary of potential benefits of polyamines on obesity-related diseases including metabolic syndrome and type 2 diabetes. Polyamines ameliorate metabolic syndrome by reducing weight gain, insulin resistance, lipid metabolism, gut barrier function, and metabolic endotoxemia. Polyamines also regulate both innate and adaptive immunity including proinflammatory cytokine production, autophagy, macrophage polarization, T cell differentiation, and B cell senescence. Further investigation is needed to elucidate polyamine-mediated health benefits in obesity-related diseases.

Together, these studies demonstrated that restoration of polyamine synthesis by *B. lactis* and *B. pseudolongum* ameliorated complications associated with obesity/T2D. Moreover, direct administration of polyamines reduced body weight, adipocyte differentiation, and lipid accumulation in obese mice (78–81). Enhancement of polyamine synthetic enzyme, SAT1, by triethylenetetramine dihydrochloride was also associated with anti-obesity and anti-diabetic effects in mice (103). There is an ongoing need to study the direct effects of polyamines on development of diabetes. In summary, these studies provide evidence that polyamines are integral to regulating metabolic syndrome and illustrate its potential as a nutraceutical for metabolic complications of obesity/T2D (**Figure 2**).

### Polyamines as a nutraceutical to improve immunity against bacterial infections in obesity/T2D

Obesity/T2D is associated with increased risk for adverse clinical outcomes post-infection likely due to deficits in the immune system that have been likened to that of aging (105). It is particularly interesting because aging has been linked to declining levels of polyamines (40, 77), chronic low-grade inflammation (termed “inflammaging”) (106), and higher morbidity and mortality during infections (107). In fact, spermidine levels are depleted in the elderly and polyamine treatment corrects autophagy and B-cell senescence (108, 109). Similar dysregulation of polyamines in obesity/T2D would affect bacteria-host immune interactions that govern infection outcomes. In preclinical studies, obesity/T2D exacerbates inflammation and infection severity in models of osteomyelitis (110, 111), bacteremia (112), and skin infections (113, 114). Although not in the context of infection, administration of polyamines or associated probiotic and prebiotics have alleviated inflammation in a variety of disease models. Treatment with *B. lactis* and arginine, an amino acid precursor to polyamine synthesis, resulted in increased levels of circulating and colonic levels of polyamines that was associated with reduced inflammatory signaling in serum of mice (67). In a model of T-cell transfer colitis, spermidine potentiated Treg differentiation and ameliorated disease pathology in the gut (94). Injection of spermine protected mice from developing acute footpad inflammation (99), which may be important for mitigating excessive inflammation in diabetic foot ulcers (114). Therefore, polyamines are emerging players in dictating bacterial-host immune interactions in obesity/T2D by regulating inflammation (**Figure 2**).

More recently, polyamines have been directly shown to reduce osteomyelitis severity in a murine model of obesity/T2D (88). Supplementation with oligofructose, a bifidogenic prebiotic, decreased *Staphylococcus aureus* burden in infected bone and tissue in obese/T2D mice. Oligofructose dampened systemic inflammatory signaling that normally exacerbates infections in obesity/T2D (111, 114, 115), consistent with prior studies (99, 116). The authors determined a 6-log fold-change in *B. pseudolongum* in the gut microbiota of obese/T2D mice due to oligofructose treatment. This compositional change was associated with elevated polyamines in the cecum and plasma of obese/T2D mice. Remarkably, direct oral administration of spermine and spermidine led to a reduction in osteomyelitis severity similar to oligofructose. These results suggest that polyamines promote beneficial effects during infections in obesity/T2D and is an unexplored area that warrants further investigation.

## Conclusion and perspectives

In this review, we propose that polyamines metabolites have the potential to ameliorate metabolic syndrome and the complications of obesity/T2D. Increasing evidence suggests that polyamines contribute to regulation of metabolic health and immunity in obesity/T2D. However, preclinical and clinical studies that examine polyamines in obesity/T2D are inconsistent due to heterogenous methods and tissue sampling. With their pleiotropic effects on transcription and translation in both eukaryotes and prokaryotes, the mechanisms by which polyamines affect human health remain unknown. In addition to the cautionary points we raise with current human data, future preclinical studies are needed to fully define the role of polyamines in obesity-associated metabolic disorders. First, obesity/T2D occurs through different mechanisms in the established diet-induced, transgenic leptin-deficient, and leptin receptor-deficient animal obesity models (117). The distinct gut microbiota and drug responses in these models affect microbiome compositional and metabolomic studies of obese/T2D animals and lean/controls (117–119). Second, the frequent use of male animals which gain weight more consistently in diet-induced murine models ignores the effect of sex on complications of obesity/T2D (120). Third, animal studies that implement probiotic, prebiotic, or post-biotics like polyamines often treat animals in parallel during the development of obesity/T2D. However, individuals with obesity/T2D often seek medical care after they have developed obesity/T2D and the complications of metabolic syndrome. Finally, previous literature has only analyzed polyamines in plasma of obese/T2D individuals, which does not consider the contributions of the gut microbiota as the primary driver of polyamine production. Further studies are needed to investigate polyamine levels in fecal samples to better understand polyamine production in obese/T2D individuals. Therefore, it is difficult to translate the results of animal experiments to personalized nutrition and precision medicine in humans.

With polyamines found as ubiquitous metabolites among eukaryotes and prokaryotes, their role in infectious disease is more complex than other microbiota-derived metabolites. Polyamines have been shown to be critical for survival and virulence of human bacterial pathogens (121, 122). While some pathogens produce polyamines, others rely on the extracellular polyamine pool regulated by host cells and the gut microbiota through uptake *via* transporter systems (121, 122) where polyamines have been shown negative effects on infectious bacteria. For example, spermine directly inhibits the growth of pathogenic E*scherichia coli, Salmonella enterica* serovar Typhimurium, and *Staphylococcus aureus*, while increasing susceptibility to β-lactam antibiotics in *Pseudomonas aeruginosa* (123). Because polyamines regulate survival and proliferation of bacteria and mammalian cells, this suggests that the host and bacteria pathogens compete for the extracellular polyamines pool during infections. For example, the polyamine transport operon *potABCD* in *Streptococcus pneumoniae* is required for resistance to neutrophil killing *in vivo* (124).This further implies a need to investigate the intracellular levels of polyamines in both mammalian cells and bacterial pathogens.

In conclusion, a growing body of evidence suggests that dysregulation of polyamines is associated with obesity/T2D and the related complications of immune deficits and metabolic disorders. The obesity/T2D epidemic is a public health concern and calls for alternative therapeutics. Probiotics, prebiotics, and post-biotics serve as mediators of metabolic syndrome and immunity in obesity/T2D. Prebiotics like oligofructose and probiotics like *Bifidobacterium* spp. that is associated polyamine production can be therapeutic alternatives to treating metabolic syndrome and strengthening of immunity, but relies on modulation of the gut microbiome. Therefore, direct application of the post-biotic, polyamines, to obese/T2D patients is a more attractive target. However, the biology of polyamines is heavily understudied outside the context cancer and remains to be investigated. Further research is needed to elucidate the mechanisms of polyamine regulation that contribute to diminished gut health, chronic inflammation in obesity, and development of diabetes, which will aid in the use of polyamines as a diagnostic tool for these complications.

## Author contributions

TB conducted the literature review and prepared the manuscript. TB, SG, GM, and EB contributed to writing and manuscript preparation. All authors contributed to the article and approved the submitted version.
